# Supplementary material for: A Humanized Yeast Phenomic Model of Deoxycytidine Kinase to Predict Genetic Buffering of Nucleoside Analog Cytotoxicity
Source: Genes (Basel). 2019 Sep 30;10(10):770. doi: 10.3390/genes10100770 (PMC6826991; doi:10.3390/genes10100770)
Supplement: Supplementary file 1 [file genes-10-00770-s001.zip › Additional_Files/Additional_File7_GOTermHeatmaps.zip!/Additional_File7_GOTermHeatmaps/endoribonuclease_activity.pdf]

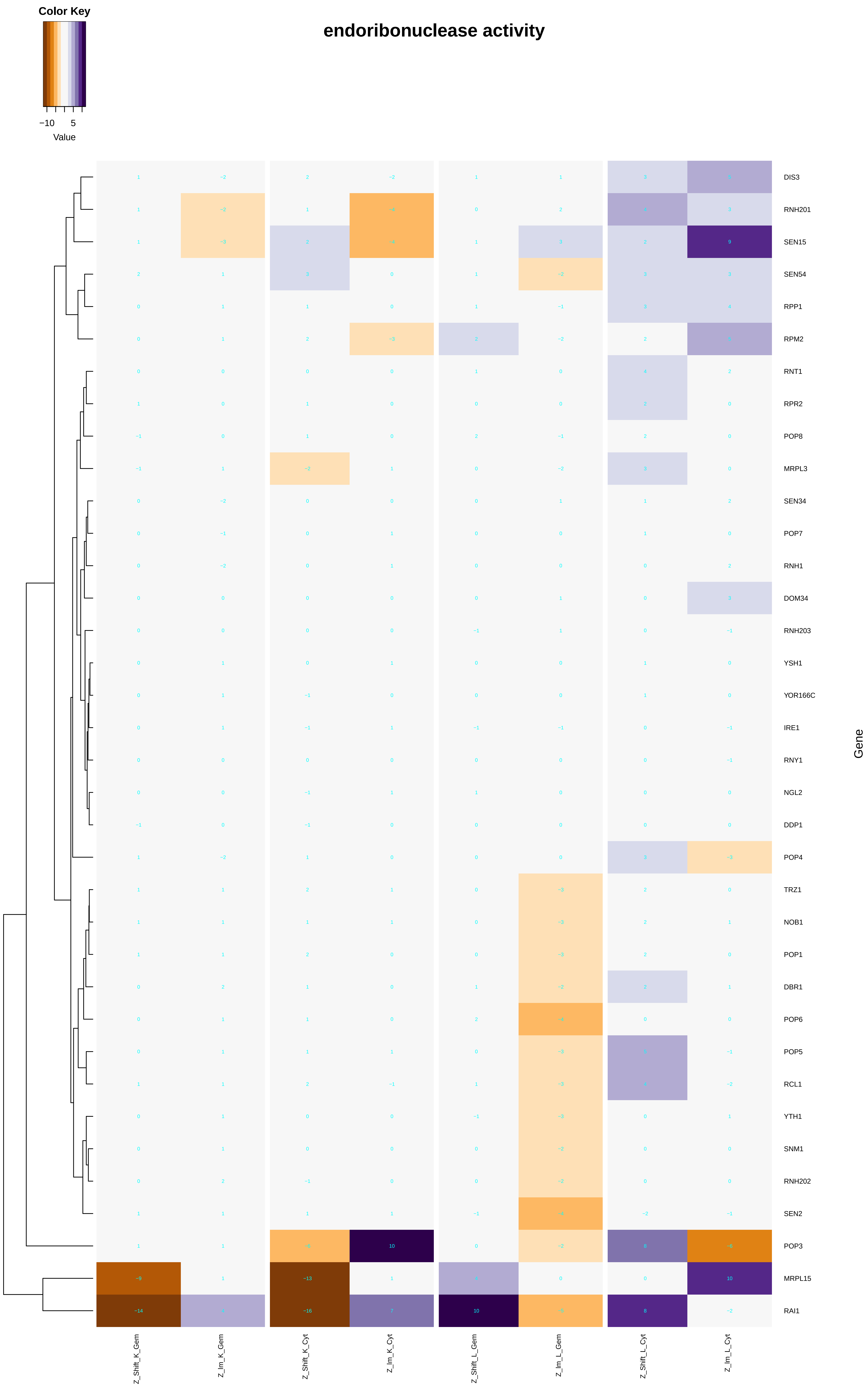
Color Key
−10
5
Value
endoribonuclease activity
DIS3
RNH201
SEN15
SEN54
RPP1
RPM2
RNT1
RPR2
POP8
MRPL3
SEN34
POP7
RNH1
DOM34
RNH203
YSH1
YOR166C
IRE1
RNY1
NGL2
DDP1
POP4
TRZ1
NOB1
POP1
DBR1
POP6
POP5
RCL1
YTH1
SNM1
RNH202
SEN2
POP3
MRPL15
RAI1
Gene
Z_Shift_K_Gem
Z_Im_K_Gem
Z_Shift_K_Cyt
Z_Im_K_Cyt
Z_Shift_L_Gem
Z_Im_L_Gem
Z_Shift_L_Cyt
Z_Im_L_Cyt

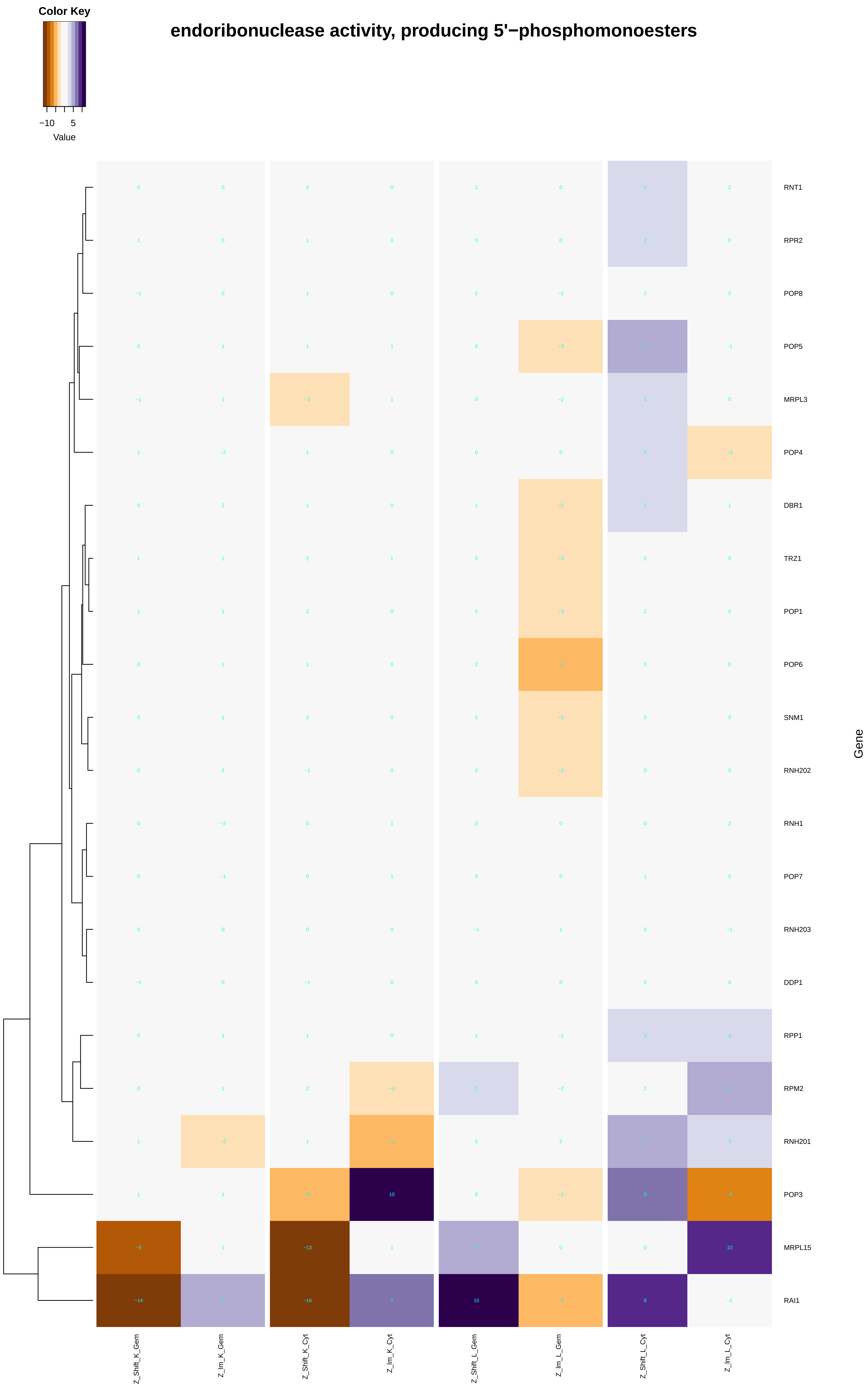
endoribonuclease activity, producing 5'−phosphomonoesters
Color Key
−10
5
Value
Gene
RNT1
RPR2
POP8
POP5
MRPL3
POP4
DBR1
TRZ1
POP1
POP6
SNM1
RNH202
RNH1
POP7
RNH203
DDP1
RPP1
RPM2
RNH201
POP3
MRPL15
RAI1
Z_Shift_K_Gem
Z_Im_K_Gem
Z_Shift_K_Cyt
Z_Im_K_Cyt
Z_Shift_L_Gem
Z_Im_L_Gem
Z_Shift_L_Cyt
Z_Im_L_Cyt

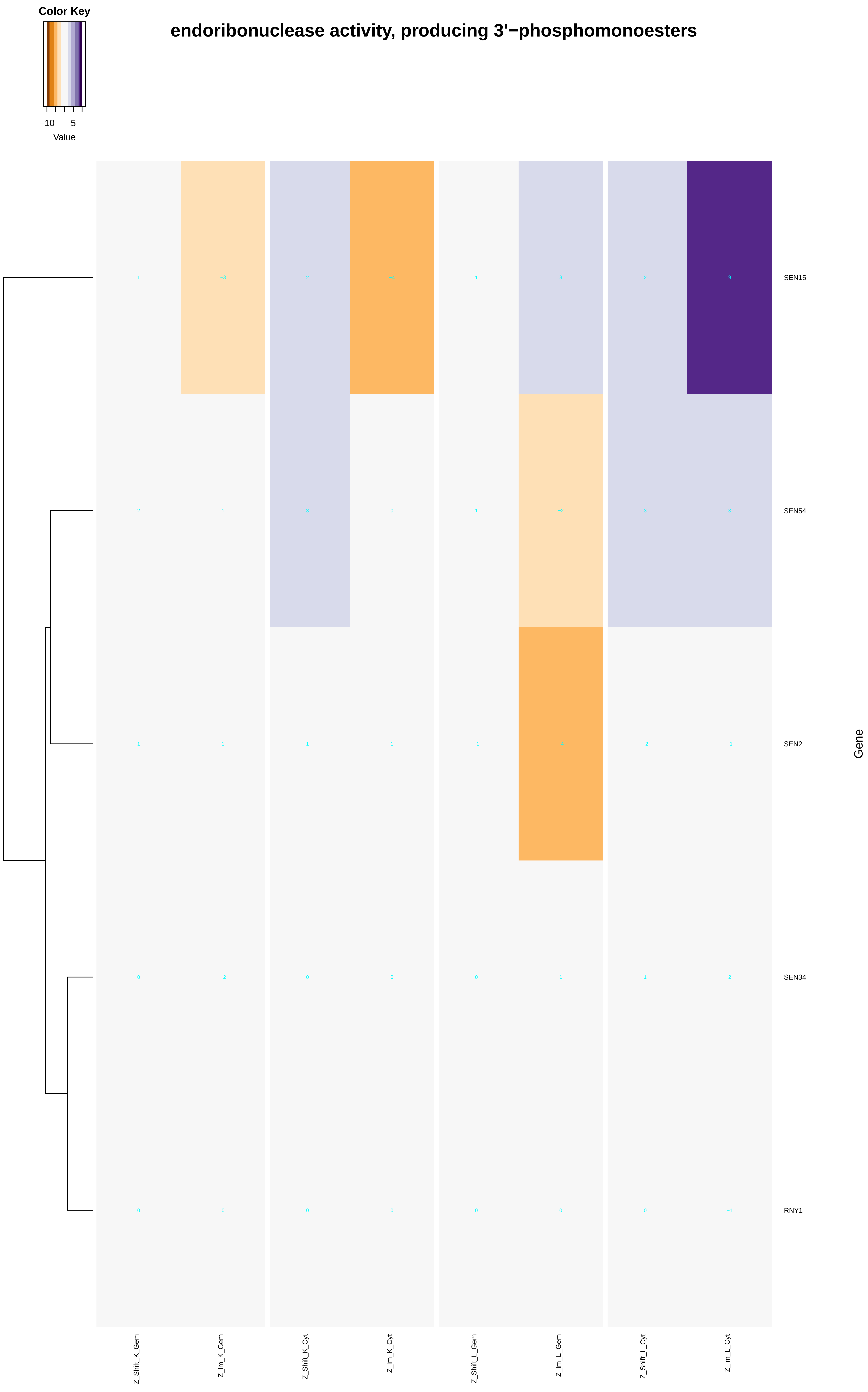
endoribonuclease activity, producing 3'−phosphomonoesters
Color Key
−10
5
Value
SEN15
SEN54
SEN2
SEN34
RNY1
Gene
Z_Shift_K_Gem
Z_Im_K_Gem
Z_Shift_K_Cyt
Z_Im_K_Cyt
Z_Shift_L_Gem
Z_Im_L_Gem
Z_Shift_L_Cyt
Z_Im_L_Cyt

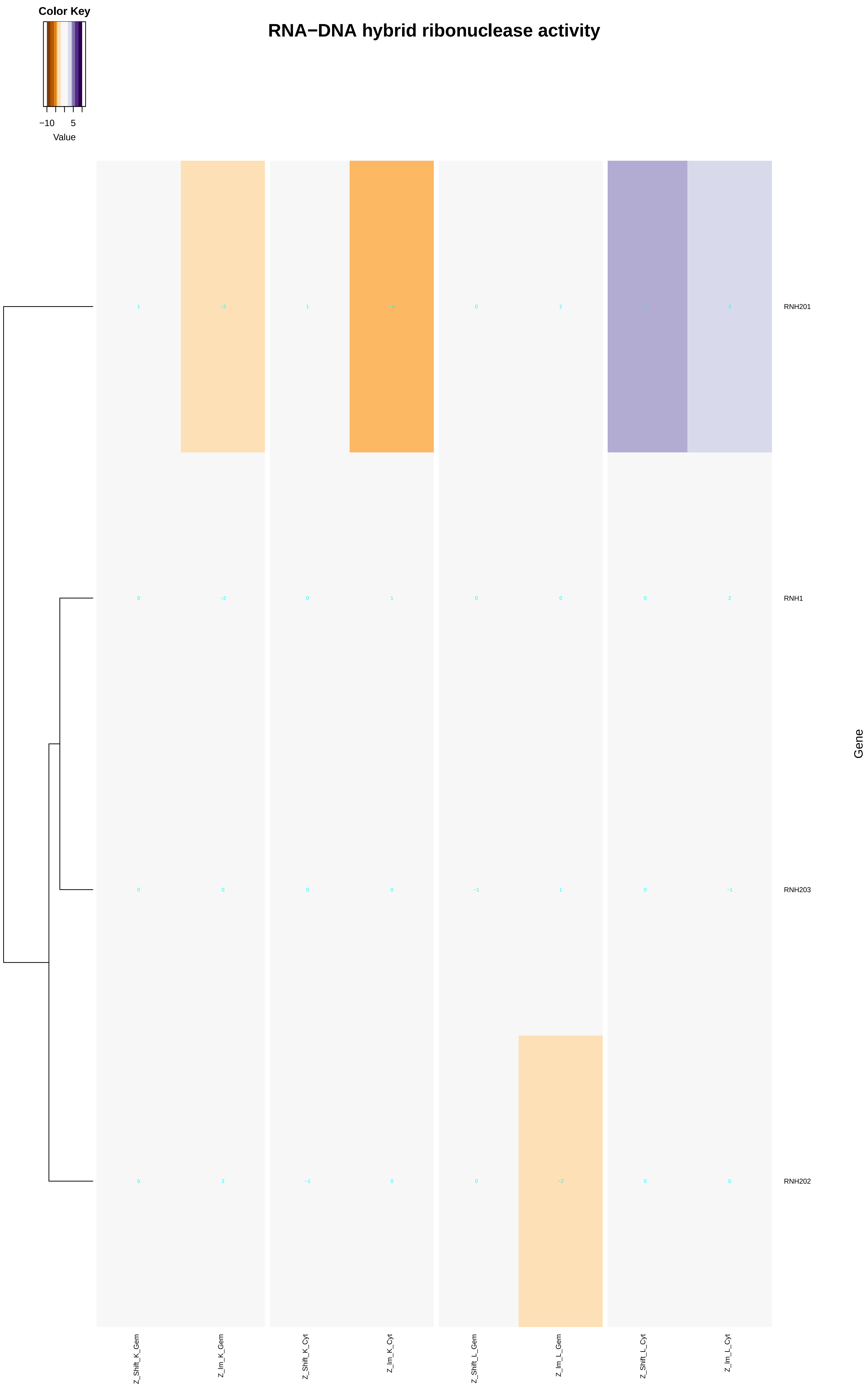

RNA−DNA hybrid ribonuclease activity
Color Key
−10
5
Value
RNH201
RNH1
RNH203
RNH202
Gene
Z_Shift_K_Gem
Z_Im_K_Gem
Z_Shift_K_Cyt
Z_Im_K_Cyt
Z_Shift_L_Gem
Z_Im_L_Gem
Z_Shift_L_Cyt
Z_Im_L_Cyt

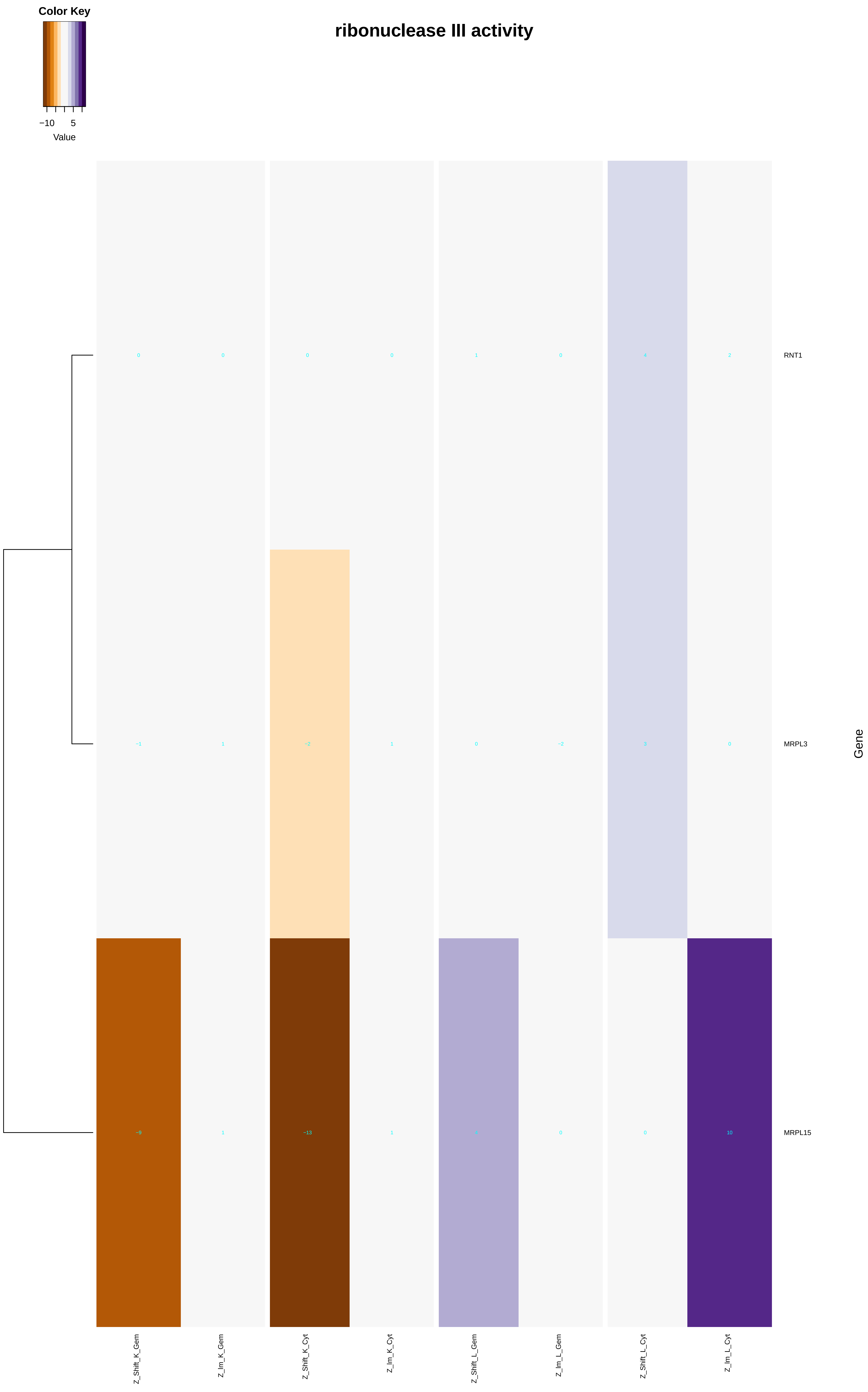
Color Key
−10
5
Value
ribonuclease III activity
RNT1
MRPL3
MRPL15
Gene
Z_Shift_K_Gem
Z_Im_K_Gem
Z_Shift_K_Cyt
Z_Im_K_Cyt
Z_Shift_L_Gem
Z_Im_L_Gem
Z_Shift_L_Cyt
Z_Im_L_Cyt

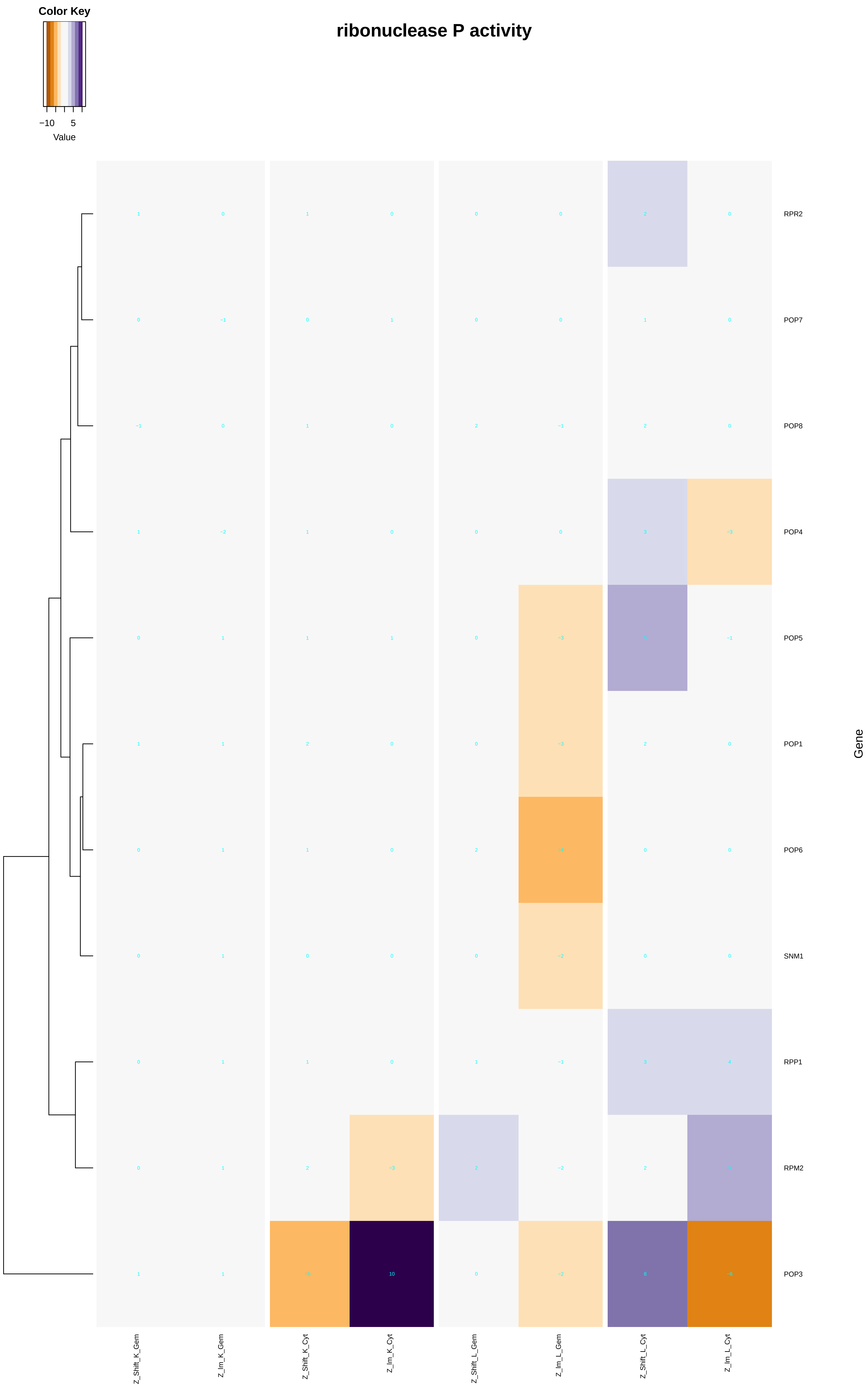
Color Key
−10
5
Value
ribonuclease P activity
RPR2
POP7
POP8
POP4
POP5
POP1
POP6
SNM1
RPP1
RPM2
POP3
Gene
Z_Shift_K_Gem
Z_Im_K_Gem
Z_Shift_K_Cyt
Z_Im_K_Cyt
Z_Shift_L_Gem
Z_Im_L_Gem
Z_Shift_L_Cyt
Z_Im_L_Cyt

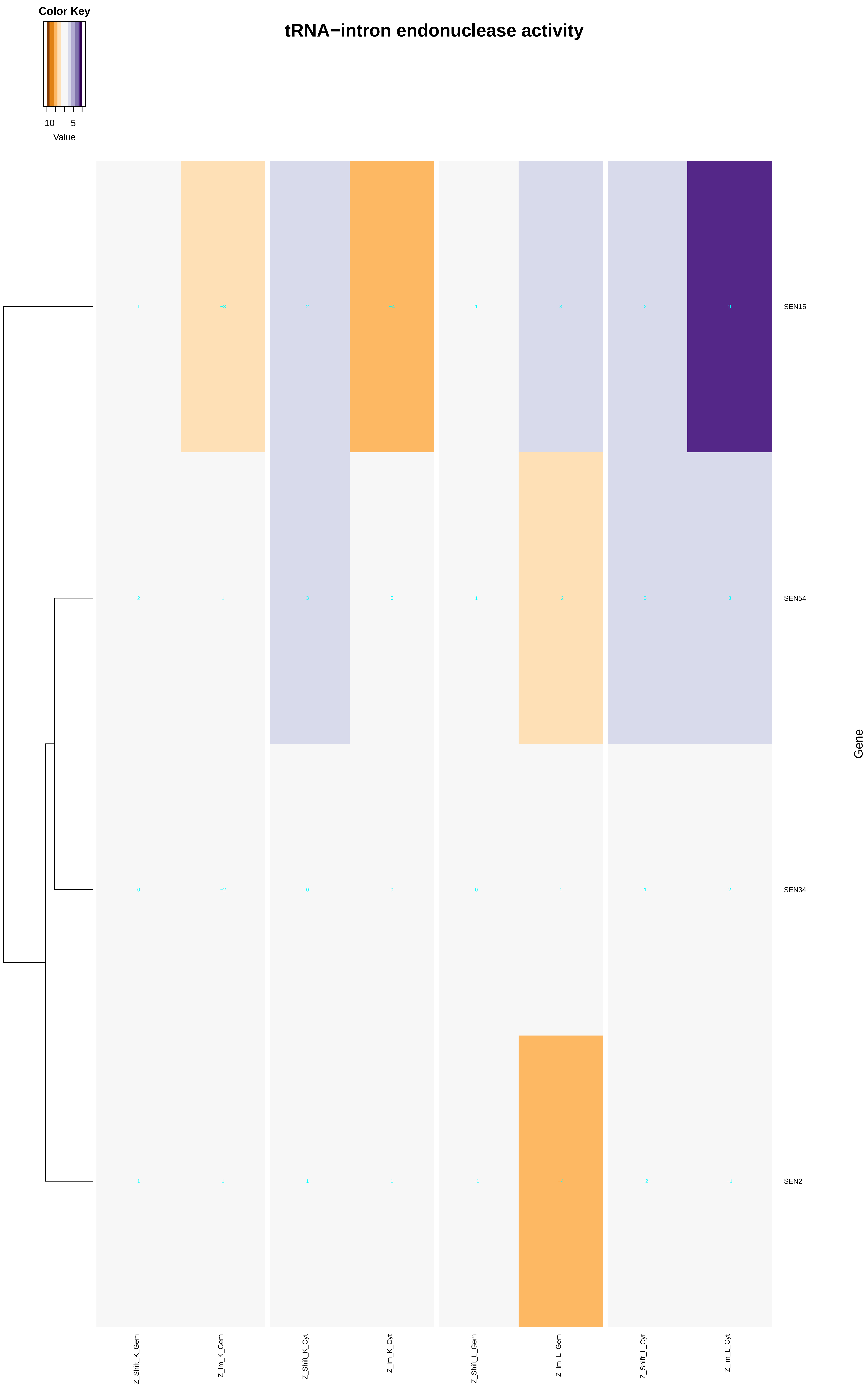

tRNA−intron endonuclease activity
Color Key
−10
5
Value
SEN15
SEN54
SEN34
SEN2
Gene
Z_Shift_K_Gem
Z_Im_K_Gem
Z_Shift_K_Cyt
Z_Im_K_Cyt
Z_Shift_L_Gem
Z_Im_L_Gem
Z_Shift_L_Cyt
Z_Im_L_Cyt
